# US State Statutes Addressing Unilateral Clinician Decisions About Life-Sustaining Treatment

**DOI:** 10.1001/jamahealthforum.2025.3508

**Published:** 2025-08-29

**Authors:** Gina M. Piscitello, Michael T. Huber, Leigh Meyer, Patrick G. Lyons, William F. Parker, Erin S. DeMartino

**Affiliations:** 1 Division of General Internal Medicine, Section of Palliative Care and Medical Ethics, University of Pittsburgh, Pittsburgh, Pennsylvania; 2 Palliative Research Center, University of Pittsburgh, Pittsburgh, Pennsylvania; 3 Division of Geriatrics and Palliative Medicine, University of Miami, Miami, Florida; 4 Institute of Bioethics and Health Policy, University of Miami, Miami, Florida; 5 Biomedical Ethics Research Program, Department of Quantitative Health Sciences, Mayo Clinic, Rochester, Minnesota; 6 Division of Pulmonary, Allergy and Critical Care, Oregon Health & Science University, Portland; 7 Department of Pulmonary and Critical Care, University of Chicago, Chicago, Illinois; 8 MacLean Center for Clinical Medical Ethics, University of Chicago, Chicago, Illinois; 9 Division of Pulmonary and Critical Care Medicine, Mayo Clinic, Rochester, Minnesota

## Abstract

This cross-sectional study evaluates US state regulation of clinician decisions about life-sustaining treatment for adults, including justifications and regulatory requirements.

## Introduction

Unilateral clinician decisions to decline initiating or maintaining life-sustaining treatment (LST) are controversial and used disproportionately for vulnerable populations.^1,2^ US hospital policies addressing these unilateral decisions rarely acknowledge these concerns.^3^ Although US state regulation of LST in specific situations (eg, resource scarcity, pregnant patients lacking decisional capacity) has been assessed, how state statutes regulate unilateral clinician decisions to decline initiating or maintaining LST during non–resource scarce periods is unknown.^4,5^

## Methods

The most current US state and District of Columbia statutes addressing decisions to decline initiating or maintaining LST for adult patients were identified by 3 authors (G.M.P, M.T.H, and L.M) using Fastcase, Casetext, and state websites from September 2024 to May 2025. This cross-sectional study followed the Strengthening the Reporting of Observational Studies in Epidemiology (STROBE) guideline. IRB review was not required per the Common Rule (US 45 CFR 46) because it did not involve human participants. G.M.P and M.T.H independently identified which statutes support unilateral clinician decisions to decline initiating or maintaining LST and regulations for these decisions. Justifications for these unilateral decisions were categorized into medical reasons (referencing a patient’s medical condition or medical standards) and reason of conscience (mentioning conscience or moral, religious, or ethical beliefs).

## Results

Among 51 statutes, 49 (96%) explicitly supported unilateral clinician decisions to decline initiating or maintaining at least 1 form of LST. Reasons justifying these decisions varied across states: 7 (14%) included medical reasons only, 9 (18%) included reasons of conscience only, 25 (49%) included both medical reasons and reasons of conscience, and 8 (16%) lacked a medical or conscience justification (Figure). Of 32 statutes permitting unilateral decisions for medical reasons, the majority 19 (59%) mentioned circumstances where providing LST was contrary to “medical standards,” followed by “medically ineffective” at 34% (n = 11), medically “inappropriate” at 28% (n = 9), “nonbeneficial” at 13% (n = 4), and/or “medically futile” at 9% (n = 3). Among 34 statutes permitting unilateral decisions for reasons of conscience, 18 (53%) mentioned the word “conscience”; 14 (41%), “moral”; 11 (32%), “religious”; and/or 8 (24%), “ethical.”

**Figure.**
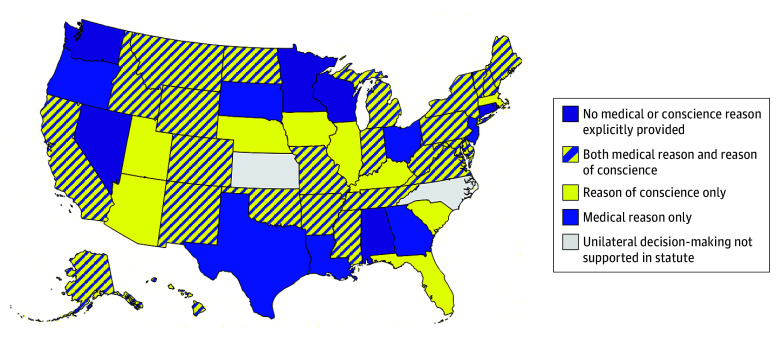
Reasons Supporting Unilateral Clinician Decisions to Decline Initiating or Maintaining Life-Sustaining Treatment Medical reason was defined as referencing a patient’s medical condition or medical standards. Reason of conscience was defined as explicit mention of conscience, or moral, religious, or ethical beliefs.

Using medical reasons to support these decisions, 56% (18 of 32) statutes required clinicians to inform the patient or surrogate of the unilateral decision, 88% (28 of 32) cooperate with transferring the patient to another clinician or institution, and 59% (19 of 32) provide LST until the patient is transferred. Indiana’s statute did not require transfer if LST was “medically inappropriate.” Four of 32 statutes (13%) allowed clinicians to decline initiating or maintaining LST if transfer could not be pursued. Only rarely did these 32 statutes require clinicians to obtain a second medical opinion (9 [28%]), pursue a medical, ethics, or interdisciplinary committee review (4 [13%]), address conflict (1 [3%]), or track and report data for patients involved in these decisions (1 [3%]). Only Texas included language forbidding use of quality of life judgments (during committee review of clinician unilateral decisions to decline initiating or maintaining LST) (Table). No statute addressed sociodemographic disparities associated with unilateral clinician decisions to decline initiating or maintaining LST.

**Table.** Required Actions If Clinicians Use Medical Reason to Decline Initiating or Maintaining Life-Sustaining Treatment

Action	States that require action
Inform patient or surrogate about decision to decline initiating or maintaining life-sustaining treatment	Alaska, Arkansas, California, Delaware, Georgia, Hawaii, Maine, Maryland, Mississippi, New Mexico, Oklahoma, Oregon, Pennsylvania, Tennessee, Texas, Vermont, Virginia, Wyoming
Obtain a second medical opinion	Alaska, Colorado, Maryland, Montana, New Hampshire, New York, Virginia, Vermont, West Virginia
Pursue a medical, ethics, or interdisciplinary committee review	Colorado, Montana, Texas,^a^ Virginia
Offer patient or surrogate the opportunity to attend medical, ethics, or interdisciplinary committee review	Texas, Virginia
Address conflict	Virginia
Cooperate with transfer to another clinician or institution	Alaska, Arkansas, California, Colorado, Georgia, Hawaii, Idaho, Louisiana, Maine, Maryland, Michigan, Mississippi, Missouri, Montana, New Jersey, New Mexico, North Dakota, Ohio, Oklahoma, Oregon, Pennsylvania, South Dakota, Tennessee, Texas, Vermont, Virginia, West Virginia, Wyoming
Do not impede transfer	Delaware
Continue to provide life-sustaining treatment until patient transferred to another clinician or institution	Alaska, Arkansas, California, Delaware, Georgia, Hawaii, Maine, Maryland, Mississippi, New Mexico, New York, North Dakota, Ohio, Oklahoma, South Dakota, Tennessee, Texas, Vermont, Wyoming
Can withhold or withdraw life-sustaining treatment if transfer cannot be pursued	Arkansas, Tennessee, Texas,^b^ Virginia^c^
Do not need to pursue transfer to another institution if life-sustaining treatment is “medically inappropriate”	Indiana
Limited to do-not-resuscitate decisions for patients who both lack decision-making capacity and a surrogate decision-maker	New Hampshire, New York
Track and report the sex, race, age, and insurance of patients without decision-making capacity brought before committees to discuss clinician decisions to decline initiating or maintaining life-sustaining treatment	Texas

^a^ In Texas, the committee’s review can consider the patient’s well-being but cannot make judgments on the patient’s quality of life.

^b^ In Texas, life-sustaining treatment can be withheld or withdrawn 25 days after a written notice is provided to patient or surrogate, unless a court grants an extension.

^c^ In Virginia, life-sustaining treatment can be stopped after 14 days, subject to the right for court review.

## Discussion

Although almost all state statutes allowed clinicians to unilaterally decline initiating or maintaining LST in some situations, the reasons supporting and actions required to pursue these decisions varied widely. For example, whereas some statutes restricted unilateral clinician decisions to patients for whom LST is “medically ineffective,” others supported clinician value judgment in decision-making (eg, for cases deemed medically “inappropriate” or “nonbeneficial” or for reasons of conscience). Only 1 state stated quality of life judgments should not be used in unilateral decisions to decline initiating or maintaining LST. Many states did not require oversight of individual clinician unilateral decisions. No statute explicitly addressed known sociodemographic disparities associated with these decisions, which might perpetuate these disparities across the US.^1,2^ One study limitation is that statutes pertaining only to pediatric patients were not assessed. Variation in statute regulation of unilateral clinician decision-making about LST might contribute differences in the provision of LST across the US and deserves further study.^6^

## Notes

Supplement.
Data Sharing Statement
