# Supplementary material for: US State Statutes Addressing Unilateral Clinician Decisions About Life-Sustaining Treatment
Source: JAMA Health Forum. 2025 Aug 29;6(8):e253508. doi: 10.1001/jamahealthforum.2025.3508 (PMC12397882; doi:10.1001/jamahealthforum.2025.3508)
Supplement: Supplement. — Data Sharing Statement [file jamahealthforum-e253508-s001.pdf]

# Data Sharing Statement

Piscitello. US State Statutes Addressing Unilateral Clinician Decisions About Life-Sustaining Treatment. *JAMA Health Forum*. Published August 29, 2025.
doi:10.1001/jamahealthforum.2025.3508

**Data**
**Data available:** Yes
**Data types:** Data (not involving human participants)
**How to access data:** Data can be accessed by emailing the corresponding author, ginapiscitello@pitt.edu
**When available:** With publication

**Supporting Documents**
**Document types:** None

**Additional Information**
**Who can access the data:** To anyone requesting the data.
**Types of analyses:** For any purpose.
**Mechanisms of data availability:** With investigator support.
